# Case Report: Neurogenic Thoracic Outlet Syndrome Without Electrophysiologic Abnormality

**DOI:** 10.3389/fneur.2021.644893

**Published:** 2021-04-09

**Authors:** Sun Woong Kim, Duk Hyun Sung

**Affiliations:** Department of Physical and Rehabilitation Medicine, Samsung Medical Center, Sungkyunkwan University School of Medicine, Seoul, South Korea

**Keywords:** neurogenic thoracic outlet syndrome, CT angiography, brachial plexus magnetic resonance imaging, medial antebrachial cutaneous nerve, case report

## Abstract

Neurogenic thoracic outlet syndrome (N-TOS) is a chronic compressive brachial plexopathy that involves the C8, T1 roots, and/or lower trunk. Medial antebrachial cutaneous (MABC) nerve conduction study (NCS) abnormality is reportedly one of the most sensitive findings among the features of N-TOS. The aim of the present study was to report clinical features, imaging findings, treatment, and prognoses of two N-TOS patients with no abnormalities in electrophysiological studies. Both patients presented with paresthesia of unilateral arm, and examination revealed no neurologic deficits. Electrophysiologic studies including MABC NCS were normal. Computed tomography (CT) angiography and brachial plexus magnetic resonance imaging (MRI) of the patients showed compression and displacement of the neurovascular bundle in the thoracic outlet by causative structures. Due to their sensory symptoms and CT angiography and brachial plexus MRI findings, after excluding other diseases, we diagnosed them with N-TOS. With the development of imaging techniques, more patients presenting with clinical features of lower trunk brachial plexopathy and anomalous structures compressing the neurovascular bundle on imaging studies can be diagnosed with N-TOS, even if electrophysiologic studies including MABC NCS do not show abnormalities.

## Introduction

Neurogenic thoracic outlet syndrome (N-TOS) is a chronic compressive brachial plexopathy that involves the C8, T1 roots, and/or lower trunk (1). In N-TOS, the brachial plexus is pressed anteriorly and upwardly by the cervical rib or fibrous band, arising elongated transverse process of C7. The disease mainly invades through the T1 root because it is located at the lowermost side of the brachial plexus. As a result, the median-innervated intrinsic hand muscles receiving T1-dominant innervation are usually involved (2). The sensory symptom also occurs mainly in the medial side of the forearm, which is medial antebrachial cutaneous (MABC) nerve territory and T1 dermatome. Within the same mechanism, MABC sensory nerve conduction study (NCS) abnormalities are reportedly the most sensitive finding among the electrophysiological features of N-TOS (3, 4). However, we recently encountered two patients with no abnormalities in electrophysiological studies, including MABC NCS, although their clinical presentations and imaging findings were compatible with N-TOS. Here, we report their clinical features, imaging findings, treatment, and prognoses.

## Case Description

### Case 1

**Figure 1:**
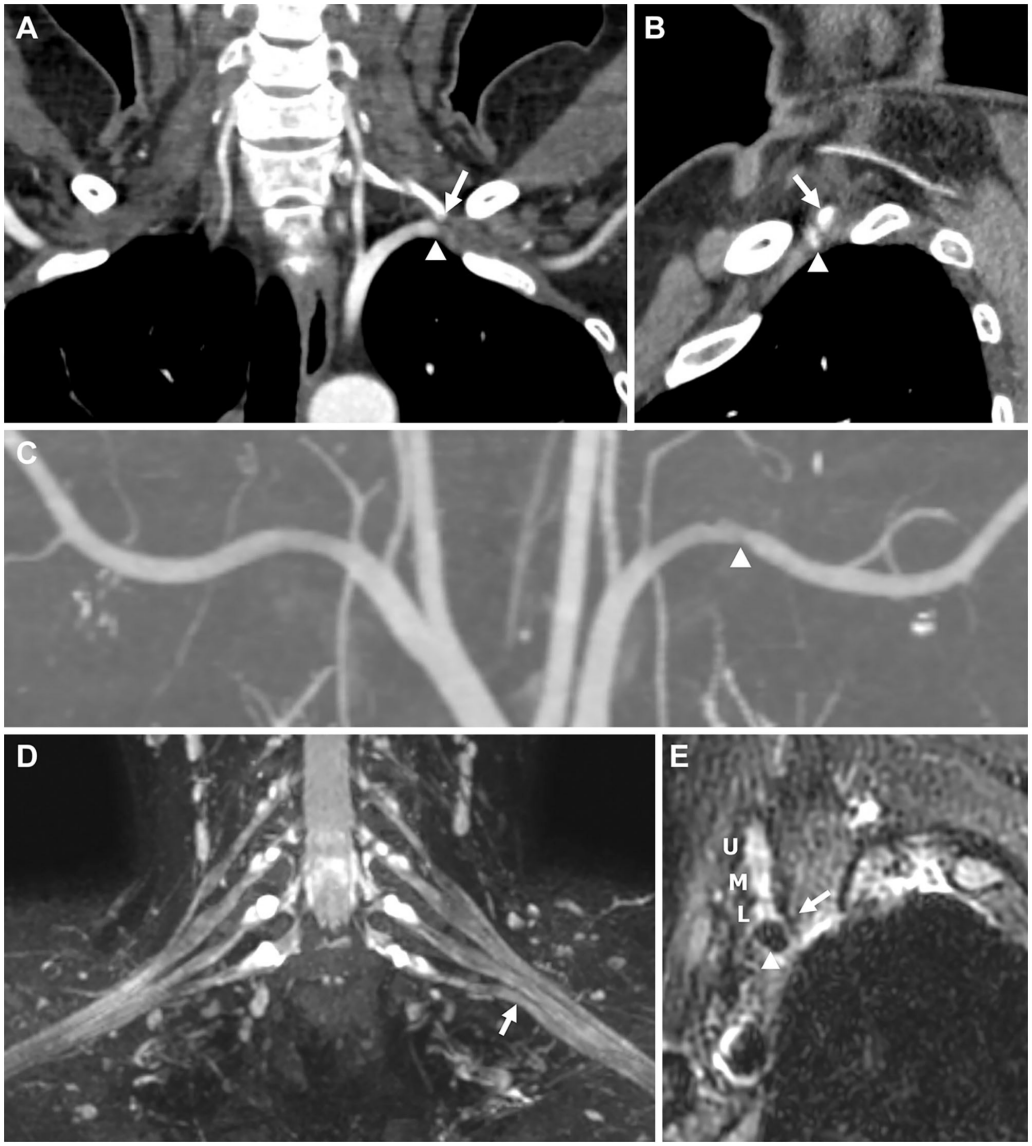
Imaging findings for Patient 1. **(A,B)** Computed tomography (CT) angiography performed with 180° shoulder abduction. **(C–E)** Brachial plexus magnetic resonance imaging (MRI). **(A)** Coronal image showing the tip of the left cervical rib arising from the C7 vertebra (arrow) abutting the left subclavian artery (arrowhead). **(B)** Sagittal image showing the tip (arrow) compressing the subclavian artery (arrowhead) in the anterior direction, causing stenosis. **(C)** Coronal maximum intensity projection (MIP) image showing stenosis of the left subclavian artery (arrowhead). **(D)** Coronal T2-weighted fat suppression MIP image showing upward angulation of the lower trunk (arrow), presumably caused by the anterior tip of the cervical rib, although not shown in this image. Swelling or abnormal signal intensity of the brachial plexus was not definite. **(E)** Oblique sagittal T2-weighted fat suppression image showing the spatial associations of the anterior tip of the cervical rib (arrow), brachial plexus (U, upper trunk; M, middle trunk; L, lower trunk), and subclavian artery (arrowhead).

A 49-year-old woman visited our locomotor pain clinic with a 4-year history of paresthesia in her left medial arm, medial forearm, and fourth and fifth fingers. Her symptoms were aggravated in the supine and left lateral decubitus positions and on rotating her head to the left side, and often woke her during sleep. The paresthesia was also aggravated when her left supraclavicular fossa area was compressed. Epidural steroid injection in the cervical spine at her local community hospital did not improve her paresthesia. Neurological examination showed no motor weaknesses or sensory deficits. Percussion of the left supraclavicular fossa provoked paresthesia of her left medial arm, medial forearm, and fourth and fifth fingers. An Adson's test was positive on her left side, showing the loss of her radial pulse. The test evaluate the reproduction of symptoms or loss of radial pulse by extending the neck and rotating the head to the symptomatic side while holding a deep inspiration. Spurling and upper extremity tension tests were negative. Sensory NCS was done in the bilateral median, ulnar and MABC nerves with the antidromic method. Median and ulnar motor NCS was also done on both sides, recorded in abductor pollicis brevis and abductor digiti minimi muscles, respectively. Age-stratified reference values derived from our electrodiagnostic laboratories were used for absolute criteria of amplitude abnormality. Needle EMG was done in limb muscles, especially the C8-T1 myotome, and cervical paraspinal muscles. These electrophysiological studies did not show any abnormalities. Computed tomography (CT) angiography of her upper extremities performed in 180° shoulder abduction showed the left cervical rib arising from the C7 vertebra and mild stenosis of the left subclavian artery near the lateral tip of the cervical rib, suggesting arterial thoracic outlet syndrome (A-TOS) (Figures 1A–C). The test showed no dissection, aneurysm, or thrombosis. Brachial plexus magnetic resonance imaging (MRI) revealed upward and anterior angulation and displacement of the left lower trunk or C8/T1 extraforaminal nerve roots of the brachial plexus, presumably caused by the anterior tip of the cervical rib (Figures 1D,E). Swelling or abnormal signal intensity of the brachial plexus were not definite. Evidence of C8 or T1 cervical radiculopathy because of neural foraminal stenosis or herniated intervertebral disc was not observed in cervical spine MRI. The results of the imaging tests indicated that the cervical rib was compressing the lower trunk/roots of the brachial plexus and the subclavian artery upwardly and anteriorly. With the sensory symptoms suggesting a lesion of the lower trunk/roots of the brachial plexus and cervical rib, along with findings of the CT angiography and brachial plexus MRI, and by excluding other diseases, we diagnosed the patient with N-TOS despite normal electrophysiological study findings. We decided to manage her conservatively because there were no ischemic symptoms/signs and focal neurologic deficits in her left upper extremities. We prescribed neuropathic pain medication (gabapentin 100 mg twice a day), which helped relieve the patient's paresthesia. The patient was trained to avoid behaviors that may cause neurovascular bundle compression, and instructed to observe if there was any weakness or numbness. We also planned to conduct imaging studies to follow the status of the subclavian artery at periodic intervals. At the last follow-up 12 months after her initial visit, although better than at initial visit, the paresthesia while lying on her left side persisted. She remained positive for Tinel's sign at the supraclavicular fossa, although weakness, atrophy, or sensory loss was not detected.

### Case 2

**Figure 2:**
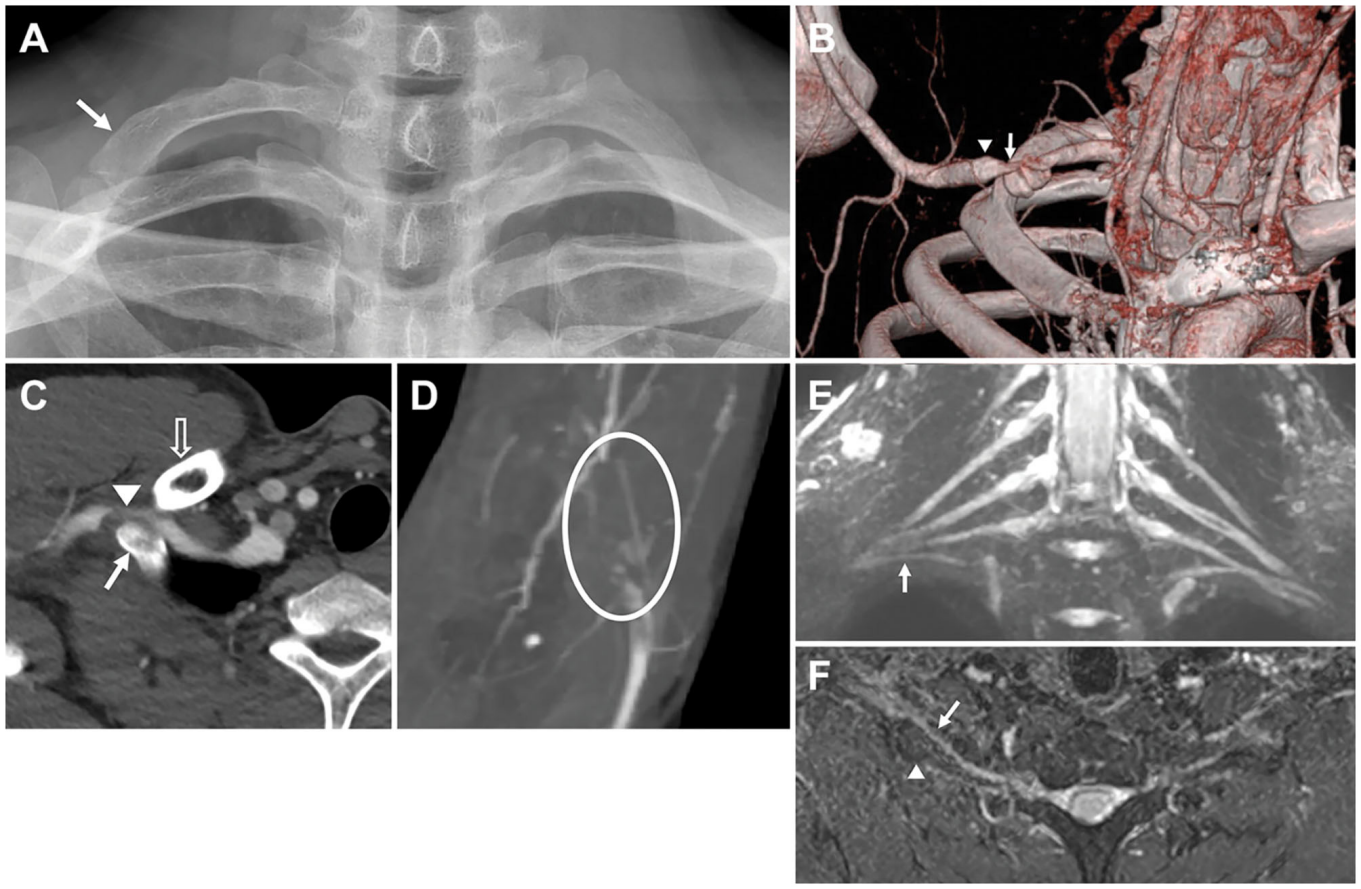
Imaging findings for Patient 2. **(B–D)** Computed tomography (CT) angiography performed with 180° shoulder abduction. **(E,F)** Brachial plexus magnetic resonance imaging (MRI). **(A)** Plain radiograph of the cervical spine showing upwardly convex curvature and an anomaly of the right first rib (arrow). **(B)** When viewed from the front, a reconstruction image using volume rendering shows stenosis (arrow) and post-stenotic dilatation (arrowhead) of the right subclavian artery. **(C)** Axial image showing the spatial relationships of the anomalous right first rib (arrow) articulating with the second rib, narrowed subclavian artery (arrowhead), and clavicle (voided arrow). **(D)** Maximum intensity projection (MIP) image showing thromboembolic occlusion of the right brachial artery at the elbow level (circle). **(E)** Coronal T2-weighted fat suppression MIP image showing a high-riding right T1 extraforaminal root and lower trunk (arrow) compared to those on the opposite side. Abnormal signal intensity of the brachial plexus was not definite. **(F)** Axial T2-weighted fat suppression MIP image showing anterior angulation of the right lower trunk (arrow) by the anomalous right first rib (arrowhead).

A 31-year-old man with a 4-month history of paresthesia on his right medial forearm and three ulnar digits was referred to our locomotor pain clinic. His paresthesia would get aggravated when lying on the right side and raising the right arm overhead for tasks, such as, when washing his hair. Neurological examination showed normal muscle strength and sensory function of his right upper extremities. No atrophy was noted in the intrinsic muscles of his right hand. Tinel's test was negative for his right supraclavicular fossa. At 90° abduction and external rotation of the shoulder (Roos test posture), the pain in his right palm and forearm worsened, and his right palm turned pale. An electrophysiological study performed at his local community hospital was reviewed by the authors. When the reference value of our institute was applied, NCS including MABC nerve did not show any abnormality. Needle EMG result was also normal. The patient's right first rib was anomalous (upwardly convex and anterior tip fused with the second rib) in cervical spine plain radiograph (Figure 2A). CT angiography of his upper extremities showed stenosis of the right subclavian artery overlying the anomalous first rib, suggesting A-TOS. The subclavian artery was dilated distal to the stenotic site. Segmental thromboembolic stenosis of the right brachial artery was also observed (Figures 2B–D). Brachial plexus MRI revealed high-riding T1 extraforaminal nerve root or lower trunk compared with that of the left side and anterior angulation of the right lower trunk by the anomalous right first rib (Figures 2E,F). Swelling or abnormal signal intensity of the brachial plexus was not definite. There was no evidence of lower cervical radiculopathy on MRI. Based on his sensory symptoms, anomalous right first rib, and CT angiography and brachial plexus MRI findings, and after excluding other diseases, we diagnosed the patient with N- and A-TOS despite normal electrophysiological study findings. The patient underwent surgical treatment (first rib resection, scalenectomy, and brachio-ulnar bypass with great saphenous vein graft) to resolve the neurovascular bundle compression and thrombosis of the brachial artery. The patient's paresthesia disappeared immediately after the operation.

## Discussion

TOS includes variable neurovascular symptoms and signs caused by compression of neurovascular bundle in the thoracic outlet. TOS is divided into three types depending on the compressed structures: arterial, venous, and neurogenic. Technical improvements in MRI is facilitating detection of brachial plexus compression in patients with N-TOS. Treatment options for N-TOS include surgery, physical therapy, behavioral modification, and pain management. It is the symptoms and physical examination findings that determines the type of treatment, rather than the presence of rib abnormalities or the results of electrophysiologic studies. Usually, initial management of N-TOS consists of conservative treatments. For patients with refractory symptoms or profound focal neurologic deficit, consideration of surgical management is needed. Optimal conservative treatment for N-TOS is still controversial. For patients with A- or V-TOS, initial intervention is most often surgical because physical therapy is not helpful (5).

Both patients described in this report showed typical neurologic symptoms of N-TOS, namely, paresthesia of the medial forearm and ulnar hand, and structural anomalies of the thoracic outlet (cervical rib and anomalous first rib). Diseases such as C8 or T1 radiculopathy, carpal tunnel syndrome, and ulnar neuropathy, which require differentiation from N-TOS, were excluded on the basis of physical examination, MRI, and electrophysiological study. It is reasonable to infer that compression of the neurovascular bundle by the structural anomalies causes neurologic symptoms. However, no neurologic deficit was observed in the neurologic examinations, and no abnormalities were observed in the electrophysiological studies. The traditional concept indicates that, because N-TOS is a slow progressing disease, patients seek medical care when overt deficits, such as weakness or atrophy of the T1 myotome muscles, occur (6). As a result, sensory abnormalities are less pronounced than weakness or atrophy (7). However, a recent study has shown that this is not the case, because more than half of the patients reported sensory symptoms as their chief complaint (3). In addition, about one-third of patients had no motor symptoms at initial presentation. Both patients in the present study also showed only sensory symptoms. Because sensory fibers are more sensitive to compression than motor fibers, it is reasonable that the sensory symptom appears early.

In electrophysiological studies, N-TOS typically reveals a characteristic “median-motor, MABC and ulnar-sensory” pattern (4, 8). The MABC is more involved than the ulnar nerve in sensory NCS because of the T1 predominance, as MABC nerve territory corresponds to the T1 dermatome and the ulnar nerve territory to the C8 dermatome (4, 9). Seror et al. (10) suggested that the MABC NCS could be used for the detection of mild lower brachial plexus lesions because they observed abnormal MABC nerve findings in all patients with unilateral, atypical pain and paresthesia of the upper limbs. Kim et al. (3) also observed MABC nerve abnormalities in all 13 patients with N-TOS and suggested that MABC NCS could be used as a screening test in patients with N-TOS because of its high sensitivity.

However, unlike previous reports, this report showed that MABC NCS is not a 100% sensitive test. In a literature review, Tsao et al. (4) described the electrodiagnostic findings of patients with N-TOS with surgical verification. Among 19 patients who underwent MABC NCS, one patient showed no abnormality. In a study of six surgically confirmed patients with N-TOS, Le Forestier et al. (8) reported one patient with normal MABC NCS. The possible explanation for the negative MABC NCS finding of N-TOS is the nature of compressive peripheral neuropathy. The pathophysiology of compressive neuropathy, such as carpal tunnel syndrome (CTS) or cubital tunnel syndrome (CuTs), is typically demyelination and may be associated with secondary axonal loss (11). Therefore, prolonged latency, reduced conduction velocity, or conduction block are usually observed initially, before amplitude reduction appears. In the case of MABC NCS, a latency delay cannot be observed because it is stimulated from the distal side of the lesion, unlike the median NCS in CTS. Therefore, patients with N-TOS in the very early stage of compressive neuropathy, before an axonal loss occurs, may not present electrophysiological abnormalities. Additionally, there are some reports that the MABC nerve is derived from both C8 and T1 dorsal root ganglia rather than T1 alone (12). In N-TOS, the T1 root is thought to receive initial compressive insult anatomically. Because of the dual supply of the MABC nerve, T1 root lesions alone may not cause abnormalities or a significant decrease of amplitude by more than 50% compared with the opposite side as the C8 root is intact.

Reports on the image features of N-TOS are scanty, mostly at the case report level, and there are no established criteria. Previous study suggested four imaging criteria for N-TOS; namely, upwardly-pushed neural structures in brachial plexus MRI, increased signal intensity of the lower roots/trunk in brachial plexus MRI, focal stenosis of the subclavian artery in CT angiography, and high-mounted subclavian artery in CT angiography (3). Because these criteria showed relatively low sensitivity (22–70%), the authors of the study recommended that imaging studies play ancillary roles in diagnosis. Baumer et al. (7) reported that only 7 of 30 patients showed abnormalities in brachial plexus MRI, which also showed low sensitivity. However, abnormalities in the imaging studies can play a definite role in diagnosis of N-TOS when there is no abnormality in electrophysiologic study. In this study, our two patients were diagnosed with N-TOS based only on the findings of imaging studies despite negative results for electrophysiologic studies including MABC NCS. Because the compressive neuropathy may progress over time and neurologic deficits or electrophysiologic abnormalities may appear later, careful monitoring of neuropathic symptoms/signs is required for patients in very early stages, especially those who had not undergone surgical decompression for causative structural abnormalities.

In conclusion, patients presenting with clinical features of lower trunk brachial plexopathy and anomalous structures compressing the neurovascular bundle on imaging studies can be diagnosed with N-TOS. The development of imaging techniques may reveal more such cases. Therefore, for these patients, brachial plexus MRI and CT angiography may be helpful to diagnose N-TOS and accompanying vascular TOS, even if electrophysiologic studies including MABC NCS do not show abnormalities.

## Data Availability Statement

The original contributions presented in the study are included in the article/Supplementary Material, further inquiries can be directed to the corresponding author/s.

## Ethics Statement

The studies involving human participants were reviewed and approved by Institutional board registry of Samsung Medical Center (registry number: 2020-08-010). Written informed consent for participation was not required for this study in accordance with the national legislation and the institutional requirements.

## Author Contributions

SK and DS: conceptualization and design of the work, acquisition, analysis, or interpretation of data, and drafting the work or revising it critically for important intellectual content. All authors have read and approved the manuscript.

## Conflict of Interest

The authors declare that the research was conducted in the absence of any commercial or financial relationships that could be construed as a potential conflict of interest.
